# A Role for PPAR*β*/*δ* in Tumor Stroma and Tumorigenesis

**DOI:** 10.1155/2008/534294

**Published:** 2008-05-15

**Authors:** Rolf Müller, Martin Kömhoff, Jeffrey M. Peters, Sabine Müller-Brüsselbach

**Affiliations:** ^1^Institute of Molecular Biology and Tumor Research (IMT), Philipps-University, Emil-Mannkopff-Strasse 2, 35032 Marburg, Germany; ^2^Department of Pediatrics, Philipps-University, Baldingerstrasse, 35043 Marburg, Germany; ^3^Department of Veterinary and Biomedical Sciences and Center for Molecular Toxicology and Carcinogenesis, 312 Life Sciences Building, The Pennsylvania State University, University Park, PA 16802-1504, USA

## Abstract

Peroxisome proliferator-activated receptor-*β*/*δ* (PPAR*β*/*δ*) is a transcription factor that is activated by endogenous fatty acid ligands and by synthetic agonists. Its role in the regulation of skeletal muscle fatty acid catabolism, glucose homeostasis, and cellular differentiation has been established in multiple studies. On the contrary, a role for PPAR*β*/*δ* in tumorigenesis is less clear because there are contradictory reports in the literature. However, the majority of these studies have not examined the role of PPAR*β*/*δ* in the tumor stroma. Recent evidence suggests that stromal PPAR*β*/*δ* regulates tumor endothelial cell proliferation and promotes differentiation leading to the properly orchestrated events required for tumor blood vessel formation. This review briefly summarizes the significance of these studies that may provide clues to help explain the reported discrepancies in the literature regarding the role of PPAR*β*/*δ* in tumorigenesis.

## 1. INTRODUCTION

Peroxisome proliferator-activated receptor-*β*/*δ* (PPAR*β*/*δ*) is a transcription factor that is activated by lipid-derived ligands [1, 2]. Major functions of PPAR*β*/*δ* are associated with the regulation of intermediary metabolism, in particular energy homeostasis, skeletal muscle lipid catabolism, and glucose metabolism [3]. PPAR*β*/*δ* is also important in the control of inflammatory responses as it modulates the function, proliferation, differentiation, and survival of immune cells, notably macrophages and lymphocytes [4]. PPAR*β*/*δ* therefore represents a highly relevant drug target for the treatment of major human diseases such as obesity, metabolic syndrome, inflammatory diseases, and arteriosclerosis, which has led to the development of several synthetic drug agonists displaying subtype selectivity and high-affinity binding [5].

Mice lacking PPAR*β*/*δ* exhibit embryonic lethality due to aberrant development and malfunction of the placenta, which is, however, modulated by the genetic background [6–8]. In line with these findings, differentiation and metabolic function of trophoblast giant cells in vitro are dependent on PPAR*β*/*δ* [8]. *Pparb* null mice also exhibit a defect in wound healing [9], and consistent with this observation, PPAR*β*/*δ* is critical for the AKT-mediated survival of keratinocytes during wound healing in skin [10]. However, in contrast to this prosurvival pathway observed in skin wound healing, PPAR*β*/*δ* also stimulates keratinocyte terminal differentiation and inhibits proliferation [6, 11–14], concomitant with a downregulation of protein kinase C and MAP kinase signaling [15]. Differentiation of the digestive tract is also regulated by PPAR*β*/*δ*, where it promotes the differentiation of Paneth cells in the intestinal crypts by downregulating the hedgehog signaling pathway [16].

## 2. PPAR*β*/*δ* AND TUMORIGENESIS

Consistent with its functional role in differentiation and proliferation, PPAR*β*/*δ* inhibits chemically induced skin carcinogenesis as enhanced skin cancer is observed in mice where PPAR*β*/*δ* has been deleted globally in all cells [17]. Since no difference in chemically induced skin carcinogenesis is observed in mice when PPAR*β*/*δ* is deleted specifically in basal keratinocytes [18], this suggests that the protective effect of PPAR*β*/*δ* in skin cancer may require functional roles in other cell types found in skin. Enhanced tumor formation has also been observed in a mouse model of Raf oncogene-induced lung adenoma formation, but the precise mechanisms and cell types involved are not known [19]. In the Apc/Min mouse lacking functional APC protein as well as in azoxymethane-induced intestinal carcinogenesis, effects of PPAR*β*/*δ* have been described for tumor growth with different outcomes. For example, one study reports that PPAR*β*/*δ* is dispensible for intestinal tumorigenesis [7], while other studies suggest that PPAR*β*/*δ* attenuates colon cancer by regulating colonocyte terminal differentiation [20–24]. Yet others suggest that PPAR*β*/*δ* potentiates colon cancer by promoting cell survival pathways [25–27]. The reason for these discrepancies, and thus the precise function of PPAR*β*/*δ* in intestinal tumor cells, remains unclear at present [28]. Importantly, none of these studies addressed the issue as to whether PPAR*β*/*δ* might play a role in cells of the tumor stoma, that is host cells recruited by the tumor, such as endothelial cells (ECs), fibroblasts and macrophages [29], and would thus add another level of complexity regarding the interpretation of results obtained with transgenic tumor mouse models. Indeed, recent work suggests that PPAR*β*/*δ* also has an essential function in the tumor stroma [30, 31], which is discussed in the following section.

## 3. A ROLE FOR PPAR*β*/*δ* IN TUMOR VASCULARIZATION

**Figure 1:**
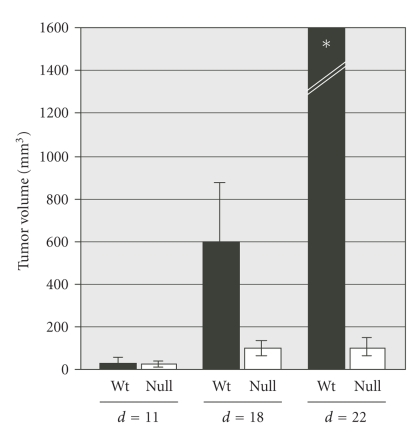
Growth of subcutaneous Lewis lung carcinoma (LLC1) in syngeneic *P* *p* *a* *r* *b* ^+/+^ and *P* *p* *a* *r* *b* ^−/−^ mice. Tumor sizes were determined at the times indicated with a caliper. The calculated volumes are shown as mean ±SD [31].*All tumor volumes <1000 mm^3^.

**Figure 2:**
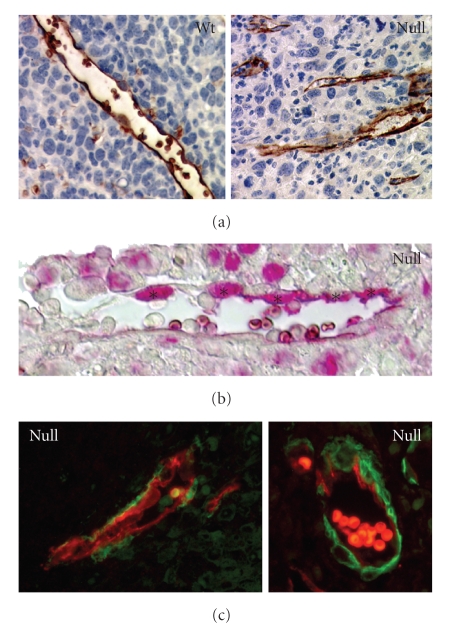
(a) Aquaporin-1 immunostaining of endothelial cells and blood vessels in subcutaneous Lewis lung carcinoma (LLC1) 14 days after inoculation into *P* *p* *a* *r* *b* ^+/+^ and *P* *p* *a* *r* *b* ^−/−^ mice (brown stain). Areas of tumor cell necrosis are obvious in the vicinity of the aberrant vascular structures in *P* *p* *a* *r* *b* ^−/−^ mice. (b) PCNA (proliferating cell nuclear antigen) staining of an LLC1 tumor section from a *P* *p* *a* *r* *b* ^−/−^ mouse. The red stain shows a high fraction of proliferating endothelial cells lining the tumor microvascular structures (denoted by asterisks; 38.7% in *P* *p* *a* *r* *b* ^−/−^ mice versus 16.6% in *P* *p* *a* *r* *b* ^+/+^ mice) [31]. (c) Aquaporin-1/*α*-smooth muscle actin double immunofluorescence of LLC1 tumors from *P* *p* *a* *r* *b* ^−/−^ mice, showing hallmarks of a hyperplastic stroma. Red: aquaporin-1, green: *α*-smooth muscle actin.

**Figure 3:**
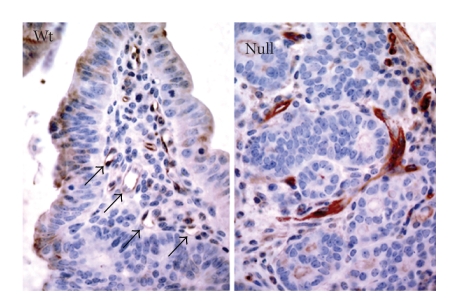
Analysis of microvessels in intestinal adenomas from *A* *P* *C* ^+/min^ mice in a *P* *P* *A* *R* *b* ^+/+^ or *P* *P* *A* *R* *b* ^−/−^ background (31 ± 3 weeks old mice) by aquaporin-1 immunostaining of paraffin sections (brown). Arrows point to normal microvessels in tumors from *P* *P* *A* *R* *b* ^+/+^ mice, lacking in *P* *P* *A* *R* *b* ^−/−^ mice. Highly aberrant vascular structures lacking a lumen are seen specifically in *P* *p* *a* *r* *b* ^−/−^ mice.

Two recent studies showed that the growth of syngeneic tumors is impaired in mice lacking PPAR*β*/*δ*. This was seen with two different subcutaneous tumor models, the Lewis lung carcinoma (LLC1) and the B16F1 melanoma [30, 31]. Tumor growth was initially indistinguishable in *Pparb* ^+/+^ and *Pparb* ^−/−^ mice, but halted after approximately 2 weeks selectively in the *P* *p* *a* *r* *b* ^−/−^ mice (Figure 1), while the inoculated *P* *p* *a* *r* *b* ^+/+^ mice invariably succumbed to their tumors within 2-3 weeks, the *P* *p* *a* *r* *b* ^−/−^ mice exhibited a survival rate of >90% after six months. Histological analyses showed that density of functional microvessels is diminished in LLC1 tumors in *P* *p* *a* *r* *b* ^−/−^ mice [30, 31]. In contrast to tumors examined in *P* *p* *a* *r* *b* ^+/+^ mice, the majority of tumor microvessels in *P* *p* *a* *r* *b* ^−/−^ mice exhibited a hyperplastic appearance typified by a thickened endothelial lining and the lack of a lumen (Figure 2(a)). Consistent with this finding, kinetic DCE-MRI analysis showed an obstructed tumor blood flow in the tumors developing in the *P* *p* *a* *r* *b* ^−/−^ mice [31]. These alterations were associated with a striking increase in tumor endothelial cell proliferation in the absence of PPAR*β*/*δ* expression (Figure 2(b)), and concomitant with this hyperproliferation, the immature ECs were surrounded by perivascular cells expressing vast amounts of the myofibroblast marker *α*-smooth muscle actin (Figure 2(c)), a picture that is characteristic of endothelial hyperplasia. These observations strongly suggest that an abnormal organization caused by a hyperplastic response, rather than a lack of ECs, underlies the abundance of abnormal microvessels in *P* *p* *a* *r* *b* ^−/−^ mice. This is consistent with a large body of evidence demonstrating that PPAR*β*/*δ* can inhibit cell proliferation in a number of different cell types [13, 24]. Importantly, PPAR*β*/*δ*-dependent tumor vascularization was not restricted to ectopic tumor models, but was also seen with intestinal adenomas in *A* *P* *C* ^+/min^ mice which showed disorganized microvessels specifically in a *P* *p* *a* *r* *b* ^−/−^ background (Figure 3). Collectively, these observations point to a general role for PPAR*β*/*δ* in the formation or maintenance of tumor blood vessels.

Although a defect in angiogenesis has not been observed during normal development of *P* *p* *a* *r* *b* ^−/−^ mice [6–9], the findings discussed above are consistent with previous findings pointing to a role for PPAR*β*/*δ* in terminal differentiation and the control of cell proliferation in different cell types, including keratinocytes [12, 14, 32, 33], trophoblast giant cells [8], and intestinal epithelial cells [16, 22]. This suggests that PPAR*β*/*δ* is specifically required by tumor ECs to orchestrate their proliferation and differentiation in an environment providing an abnormally rich source of growth factors and cytokines. A role for PPAR*β*/*δ* in tumor vascularization is also supported by several pieces of circumstantial evidence: *Pparb* is the predominant *Ppar* subtype expressed in mouse and human tumor endothelial cells, and it is upregulated by angiogenic growth factors of the tumor microenvironment [30, 31].

## 4. PPAR*β*/*δ* TARGET GENES RELEVANT FOR STROMA CELL FUNCTION

Microarray and qPCR analysis led to the identification of a set of genes that are differentially expressed in an in vivo model of growth factor-induced angiogenesis (matrigel plugs) from *P* *p* *a* *r* *b* ^+/+^ and *P* *p* *a* *r* *b* ^−/−^ mice [31]. Consistent with the observed hyperproliferative phenotype in *P* *p* *a* *r* *b* ^−/−^ mice, three of these genes have known inhibitory functions in angiogenesis (Cd36, Thbs2) or cell cycle control (Cdkn1c) [34, 35]. Thrombospondins attenuate EC proliferation and migrationin vitro and inhibit angiogenesis in vivo, which is strictly dependent on their interaction with the CD36 receptor. In *P* *P* *A* *R* *b* ^−/−^ cells, both ligand (Thbs2) and receptor(Cd36) genes are downregulated, suggesting that an autocrine or paracrine signaling loop with an essential function in modulating angiogenesis is impaired in these cells. Very little is known about the intracellular events that occur after binding of thrombospondin to CD36, so it is difficult to speculate at present about the CD36-triggered signal transduction pathway(s) that is/are affected in ECs lacking PPAR*β*/*δ* expression. The third gene identified as a PPAR*β*/*δ* target gene in this context is *Cdkn1c* [31], which codes for the CIP/KIP family member p57^KIP2^ that it is likely to function as a cyclin-dependent kinase inhibitor [34]. Thus, p57^KIP2^ would have a similar effect on EC proliferation as CD36 and thrombospondin, suggesting that these molecules may act in concert. It is likely that additional genes with functions in growth control and differentiation will be identified as potential PPAR*β*/*δ* target genes in the same experimental system, and it is likely that multiple PPAR*β*/*δ* regulated genes are important in the context of tumor stroma development and tumor angiogenesis.

## 5. CONCLUSIONS

The findings discussed above are consistent with a model where PPAR*β*/*δ* is required to modulate the angiogenic response to growth factors during the final stages of tumor angiogenesis, which is characterized by an inhibition of EC proliferation and the acquisition of a fully differentiated phenotype [36]. The lack of PPAR*β*/*δ* with the ensuing decreased expression of negative regulators of proliferation may result in a deregulation of angiogenesis with the consequence of tumor endothelial hyperplasia. A similar phenotype of enhanced, but nonproductive, angiogenesis has very recently been described in mice lacking the Notch ligand Delta-Like 4 (Dll4) [37, 38]. In contrast to PPAR*β*/*δ*, however, Dll4 is essential not only for tumor angiogenesis but also for embryonic vascular development and arteriogenesis [39], and there seems to be no cross-talk or interaction between both the PPAR*β*/*δ* and Notch/Dll4 pathways. This suggests that multiple and presumably mutually independent regulatory mechanisms are required to prevent the deregulation of tumor EC proliferation and the occurrence of nonproductive angiogenesis. The current evidence suggests that PPAR*β*/*δ* is such a regulator.

Previous studies addressing the role of PPAR*β*/*δ* in tumorigenesis have yielded partly conflicting results leaving it unclear whether PPAR*β*/*δ* has tumor-promoting or suppressing properties, in particular in colon cancer models (reviewed in [28]). Our findings provide some insight that may eventually help to resolve this issue. PPAR*β*/*δ* may have different functions in tumor stroma and in certain tumor cells with opposing effects on tumor growth. Clearly, a detailed understanding of these complexities will be a prerequisite for the development of PPAR*β*/*δ* directed drugs and their clinical application.

## References

[1] Molnár F, Matilainen M, Carlberg C (2005). Structural determinants of the agonist-independent association of human peroxisome proliferator-activated receptors with coactivators. *Journal of Biological Chemistry*.

[2] Michalik L, Auwerx J, Berger JP (2006). International union of pharmacology. LXI. Peroxisome proliferator-activated receptors. *Pharmacological Reviews*.

[3] Desvergne B, Michalik L, Wahli W (2006). Transcriptional regulation of metabolism. *Physiological Reviews*.

[4] Kostadinova R, Wahli W, Michalik L (2005). PPARs in diseases: control mechanisms of inflammation. *Current Medicinal Chemistry*.

[5] Peraza MA, Burdick AD, Marin HE, Gonzalez FJ, Peters JM (2006). The toxicology of ligands for peroxisome proliferator-activated receptors (PPAR). *Toxicological Sciences*.

[6] Peters JM, Lee SST, Li W (2000). Growths, adipose, brain, and skin alterations resulting from targeted disruption of the mouse peroxisome proliferator-activated receptor *β*(*δ*). *Molecular and Cellular Biology*.

[7] Barak Y, Liao D, He W (2002). Effects of peroxisome proliferator-activated receptor *δ* on placentation, adiposity, and colorectal cancer. *Proceedings of the National Academy of Sciences of the United States of America*.

[8] Nadra K, Anghel SI, Joye E (2006). Differentiation of trophoblast giant cells and their metabolic functions are dependent on peroxisome proliferator-activated receptor *β*/*δ*. *Molecular and Cellular Biology*.

[9] Michalik L, Desvergne B, Tan NS (2001). Impaired skin wound healing in peroxisome proliferator-activated receptor (PPAR)*α* and PPAR*β* mutant mice. *Journal of Cell Biology*.

[10] Di-Poï N, Tan NS, Michalik L, Wahli W, Desvergne B (2002). Antiapoptotic role of PPAR*β* in keratinocytes via transcriptional control of the Akt1 signaling pathway. *Molecular Cell*.

[11] Westergaard M, Henningsen J, Svendsen ML (2001). Modulation of keratinocyte gene expression and differentiation by PPAR-selective ligands and tetradecylthioacetic acid. *Journal of Investigative Dermatology*.

[12] Schmuth M, Haqq CM, Cairns WJ (2004). Peroxisome proliferator-activated receptor (PPAR)-*β*/*δ* stimulates differentiation and lipid accumulation in keratinocytes. *Journal of Investigative Dermatology*.

[13] Burdick AD, Kim DJ, Peraza MA, Gonzalez FJ, Peters JM (2006). The role of peroxisome proliferator-activated receptor-*β*/*δ* in epithelial cell growth and differentiation. *Cellular Signalling*.

[14] Kim DJ, Bility MT, Billin AN, Willson TM, Gonzalez FJ, Peters JM (2006). PPAR*β*/*δ* selectively induces differentiation and inhibits cell proliferation. *Cell Death & Differentiation*.

[15] Kim DJ, Murray IA, Burns AM, Gonzalez FJ, Perdew GH, Peters JM (2005). Peroxisome proliferator-activated receptor-*β*/*δ* inhibits epidermal cell proliferation by down-regulation of kinase activity. *Journal of Biological Chemistry*.

[16] Varnat F, Heggeler BB, Grisel P (2006). PPAR*β*/*δ* regulates paneth cell differentiation via controlling the hedgehog signaling pathway. *Gastroenterology*.

[17] Kim DJ, Akiyama TE, Harman FS (2004). Peroxisome proliferator-activated receptor *β* (*δ*)-dependent regulation of ubiquatin C expression contributes to attenuation of skin carcinogenesis. *Journal of Biological Chemistry*.

[18] Indra AK, Castaneda E, Antal MC (2007). Malignant transformation of DMBA/TPA-induced papillomas and nevi in the skin of mice selectively lacking retinoid-X-receptor *α* in epidermal keratinocytes. *Journal of Investigative Dermatology*.

[19] Müller-Brüsselbach S, Ebrahimsade S, Jäkel J (2007). Growth of transgenic RAF-induced lung adenomas is increased in mice with a disrupted PPAR*β*/*δ* gene. *International Journal of Oncology*.

[20] Harman FS, Nicol CJ, Marin HE, Ward JM, Gonzalez FJ, Peters JM (2004). Peroxisome proliferator-activated receptor-*δ* attenuates colon carcinogenesis. *Nature Medicine*.

[21] Reed KR, Sansom OJ, Hayes AJ (2004). PPAR*δ* status and Apc-mediated tumourigenesis in the mouse intestine. *Oncogene*.

[22] Marin HE, Peraza MA, Billin AN (2006). Ligand activation of peroxisome proliferator-activated receptor *β* inhibits colon carcinogenesis. *Cancer Research*.

[23] Hollingshead HE, Killins RL, Borland MG (2007). Peroxisome proliferator-activated receptor-*β*/*δ* (PPAR*β*/*δ*) ligands do not potentiate growth of human cancer cell lines. *Carcinogenesis*.

[24] Hollingshead HE, Borland MG, Billin AN, Willson TM, Gonzalez FJ, Peters JM (2008). Ligand activation of peroxisome proliferator-activated receptor-*β*/*δ* (PPAR*β*/*δ*) and inhibition of cyclooxygenase 2 (COX2) attenuate colon carcinogenesis through independent signaling mechanisms. *Carcinogenesis*.

[25] Gupta RA, Wang D, Katkuri S, Wang H, Dey SK, DuBois RN (2004). Activation of nuclear hormone receptor peroxisome proliferator-activated receptor-*δ* accelerates intestinal adenoma growth. *Nature Medicine*.

[26] Wang D, Wang H, Shi Q (2004). Prostaglandin E_2_ promotes colorectal adenoma growth via transactivation of the nuclear peroxisome proliferator-activated receptor *δ*. *Cancer Cell*.

[27] Wang D, Wang H, Guo Y (2006). Crosstalk between peroxisome proliferator-activated receptor *δ* and VEGF stimulates cancer progression. *Proceedings of the National Academy of Sciences of the United States of America*.

[28] Peters JM, Hollingshead HE, Gonzalez FJ Role of peroxisome proliferator-activated receptor-*β*/*δ* (PPAR*β*/*δ*) in gastrointestinal tract function and disease.

[29] Bissell MJ, Radisky D (2001). Putting tumours in context. *Nature Reviews Cancer*.

[30] Abdollahi A, Schwager C, Kleeff J (2007). Transcriptional network governing the angiogenic switch in human pancreatic cancer. *Proceedings of the National Academy of Sciences of the United States of America*.

[31] Müller-Brüsselbach S, Kömhoff M, Rieck M (2007). Deregulation of tumor angiogenesis and blockade of tumor growth in PPAR*β*-deficient mice. *The EMBO Journal*.

[32] Tan NS, Michalik L, Noy N (2001). Critical roles of PPAR*β*/*δ* in keratinocyte response to inflammation. *Genes & Development*.

[33] Burdick AD, Bility MT, Girroir EE (2007). Ligand activation of peroxisome proliferator-activated receptor-*β*/*δ* (PPAR*β*/*δ*) inhibits cell growth of human N/TERT-1 keratinocytes. *Cellular Signalling*.

[34] Lee M-H, Reynisdóttir I, Massagué J (1995). Cloning of p57^*K**I**P*2^, a cyclin-dependent kinase inhibitor with unique domain structure and tissue distribution. *Genes & Development*.

[35] Armstrong LC, Bornstein P (2003). Thrombospondins 1 and 2 function as inhibitors of angiogenesis. *Matrix Biology*.

[36] Carmeliet P (2000). Mechanisms of angiogenesis and arteriogenesis. *Nature Medicine*.

[37] Noguera-Troise I, Daly C, Papadopoulos NJ (2006). Blockade of Dll4 inhibits tumour growth by promoting non-productive angiogenesis. *Nature*.

[38] Ridgway J, Zhang G, Wu Y (2006). Inhibition of Dll4 signalling inhibits tumour growth by deregulating angiogenesis. *Nature*.

[39] Krebs LT, Shutter JR, Tanigaki K, Honjo T, Stark KL, Gridley T (2004). Haploinsufficient lethality and formation of arteriovenous malformations in Notch pathway mutants. *Genes & Development*.

